# The role of carboxy-terminal cross-linking telopeptide of type I collagen, dual x-ray absorptiometry bone strain and Romberg test in a new osteoporotic fracture risk evaluation: A proposal from an observational study

**DOI:** 10.1371/journal.pone.0190477

**Published:** 2018-01-05

**Authors:** Fabio M. Ulivieri, Luca P. Piodi, Enzo Grossi, Luca Rinaudo, Carmelo Messina, Anna P. Tassi, Marcello Filopanti, Anna Tirelli, Francesco Sardanelli

**Affiliations:** 1 Nuclear Medicine Unit, Fondazione IRCCS Cà Granda Ospedale Maggiore Policlinico, Milan, Italy; 2 Gastroenterology and Digestive Endoscopy Unit, Fondazione IRCCS Cà Granda Ospedale Maggiore Policlinico, Milan, Italy; 3 Villa Santa Maria Institute, Tavernerio (CO), Italy; 4 TECHNOLOGIC S.r.l. Hologic Italia, Turin, Italy; 5 Postgraduation School in Radiodiagnostics, University of Milan, Milan, Italy; 6 Physical Medicine and Rehabilitation Physician, A.S.P. I.M.M e S. e P.A.T, Milan, Italy; 7 Endocrinology and Metabolic Diseases Unit, Fondazione IRCCS Ca' Granda Ospedale Maggiore Policlinico, Milan, Italy; 8 Clinical Chemistry and Microbiology Laboratory, Fondazione IRCCS Ca' Granda Ospedale Maggiore Policlinico, Milan, Italy; 9 Radiodiagnostics Unit, IRCCS Policlinico San Donato, Milan, Italy; Seoul National University College of Medicine, REPUBLIC OF KOREA

## Abstract

The consolidated way of diagnosing and treating osteoporosis in order to prevent fragility fractures has recently been questioned by some papers, which complained of overdiagnosis and consequent overtreatment of this pathology with underestimating other causes of the fragility fractures, like falls. A new clinical approach is proposed for identifying the subgroup of patients prone to fragility fractures.

This retrospective observational study was conducted from January to June 2015 at the Nuclear Medicine-Bone Metabolic Unit of the of the Fondazione IRCCS Ca' Granda, Milan, Italy. An Italian population of 125 consecutive postmenopausal women was investigated for bone quantity and bone quality. Patients with neurological diseases regarding balance and vestibular dysfunction, sarcopenia, past or current history of diseases and use of drugs known to affect bone metabolism were excluded. Dual X-ray absorptiometry was used to assess bone quantity (bone mineral density) and bone quality (trabecular bone score and bone strain). Biochemical markers of bone turnover (type I collagen carboxy-terminal telopeptide, alkaline phosphatase, vitamin D) have been measured. Morphometric fractures have been searched by spine radiography. Balance was evaluated by the Romberg test. The data were evaluated with the neural network analysis using the Auto Contractive Map algorithm. The resulting semantic map shows the Minimal Spanning Tree and the Maximally Regular Graph of the interrelations between bone status parameters, balance conditions and fractures of the studied population. A low fracture risk seems to be related to a low carboxy-terminal cross-linking telopeptide of type I collagen level, whereas a positive Romberg test, together with compromised bone trabecular microarchitecture DXA parameters, appears to be strictly connected with fragility fractures. A simple assessment of the risk of fragility fracture is proposed in order to identify those frail patients at risk for osteoporotic fractures, who may have the best benefit from a pharmacological and physiotherapeutic approach.

## Introduction

Osteoporosis (OP) is a pathological condition in which a reduction in bone mass and an impairment of microarchitecture are found. The consequence is a decrease in bone strength, which is the result of the sum of good bone quantity and quality, followed by an increase in bone fragility [1,2]. Fractures lead to high rates of disability and mortality with high social costs. It has been calculated that, among women’s deaths associated with fractures, about 50% are due to hip fractures, 28% to vertebral fractures and 22% to fractures in other sites[1–4]. Moreover, vertebral fractures, which are the most frequent but less dangerous osteoporotic fractures, are directly related to a high risk of subsequent fractures[5,6].

Diagnosis of OP and follow-up of its treatment are performed by dual X-ray photon absorptiometry (DXA), measuring areal bone mineral density (aBMD), which is considered the most accurate diagnosing method, being aBMD the main parameter reflecting bone strength[7]. Following the indications of International Societies [8], the densitometric scans are obtained from lumbar spine and proximal femur, which are the bone segments more frequently affected by osteoporotic “fragility” fractures, that means occurring with minimal or no trauma at all. The drawn aBMD value is expressed as T-score (standard deviation from the normal reference population) and Z-score (standard deviation from the sex and age matched population). Recently, a new tool derived from DXA by a specific software has been developed, called trabecular bone score (TBS), which is able through a numerical value to give insight to the microarchitectural condition of vertebral bone. Studies have shown its ability to predict the risk of fragility fractures in OP also without relation to BMD[9–20].

The amelioration of bone strength is the aim of the pharmacological treatment of OP, which acts by reducing bone tissue resorption and/or augmenting bone tissue formation [1]. OP therapy is expensive and of long duration, so tools for fracture risk calculation have been developed, such as QFracture, Garvan fracture risk calculator, FRAX (fracture risk assessment tool), which is the most used worldwide [21,22].

Recently some papers[23–25] have questioned the common point of view about the clinical approach to diagnosis and treatment of osteoporosis, which is consolidated in the guidelines of the principal scientific societies. The most relevant criticisms are the following: first. Fewer than one in three hip fractures are attributable to bone fragility. Fractures are traumatic events caused by falls, mostly in oldest age, when fracture incidence increases dramatically. Falls and not osteoporosis would be the principal cause of fractures.

Second. The great majority of trials about the effects of pharmacological treatment for osteoporosis on fracture risk deals with a population younger than that in which most fractures occur. Third. The goal of treatment is fracture prevention and for this purpose tools have been developed to estimate the individual absolute risk for major osteoporotic fractures, in order to improve the identification of patients who would get a benefit from pharmacological treatment. However, the current FRAX based thresholds of NOGG (National Osteoporosis Guidelines Group) and NOF (National Osteoporosis Foundation) for treatment appear to advise therapy of patients not really necessitating it. Fourth. Little importance is given to individual frailty, like impaired balance, as a cause of falls and consequent fractures, and therefore this clinical condition is underdiagnosed and undertreated.

These criticisms have raised a hot debate[26–31], and most of the cited arguments have been countered on the basis of the existing literature, without reaching a definitive clarification of the question.

In this study of a postmenopausal population, with the help of the ANN analysis of many parameters regarding fracture risk, a proposal is given for the identification of the patients who may gain the greatest profit from the treatment, in order to avoid osteoporosis fragility fractures.

## Patients and methods

### Patients

This retrospective observational study was conducted from January to June 2015 at the Bone Metabolic Unit of the Nuclear Medicine of the Fondazione IRCCS Ca' Granda-Ospedale Maggiore Policlinico, Milan, Italy. One hundred and twenty-five consecutive postmenopausal caucasian women, spontaneously afferent to the Unit for a first clinical evaluation, were recruited. The exclusion criteria were past or current history of diseases and drugs known to affect bone metabolism. Moreover, patients affected by neurological diseases, affecting control of balance were excluded, as well as clinical conditions impairing balance such as orthostatic hypotension and evident sarcopenia (S1 File).

All subjects had given their written witnessed general informed consent for data scientific management, accordingly to the statement of the Hospital which is in accordance with the Helsinki Declaration II.

This study is a subsequent analysis of clinical data of a study approved by the local Ethical Committee Milano Area B (N.2421, 16^th^ October 2012). The committee reviewed the current study and declared that it was exempt from the requirement of ethical approval, being an ancillary study of pre-existing data.

### Bone quantity assessment

Bone mineral density was measured by dual-energy X-ray absorptiometry (Hologic Discovery A, Waltham, MA, USA) at lumbar spine (LS, *in vivo* precision less than 1.0%), at total and femoral neck (TN and FN, *in vivo* precision less than 2.3% and 1.8%, respectively). Individual aBMD values were expressed as SD units (T-scores and Z-scores) in relation to the reference population provided by the manufacturer. Fractured vertebrae were excluded from aBMD measurement. Osteoporosis was defined on the basis of a BMD T-score ≤ -2.5 at any site[32].

### Bone quality assessment

In all patients TBS was assessed in the region of LS-BMD DXA. As described in detail previously[15], TBS is a bone textural measure obtained from the lumbar spine DXA scan, which is able to evaluate the bone trabecular microarchitecture[9,33–39]. A mathematical model called Finite Element Method (FEM)[40,41] was used to calculate bone strain (BS) from lumbar spine DXA scans. BS represents the average strain calculated within the vertebra of the lumbar spine. The force acting on the surface of each vertebra has been calculated on the base of height and weight specific for each patient, and then a classical approach of FEM has been applied defining the stiffness matrix dependent from the local BMD[42,43]. The result of this process is the distribution of the strain defined as the spatial deformation of every single element in which the vertebra was divided before the calculation.

Serum samples were collected in all patients to measure: alkaline phosphatase total activity (ALP) by colorimetric method (Modular, Roche); its bone isoenzyme (BAP) by semiquantitative electrophoretic method; 25OH vitamin D by chemiluminescent assay (Liaison, Diasorin); carboxy-terminal cross-linking telopeptide of type I collagen (CTX) by the Serum Cross-Laps One Step ELISA method (Modular, Roche).

### Spine fracture assessment

A conventional spinal radiograph in lateral and anteroposterior projection (T4–L4) was obtained in all subjects. Two physicians, blinded to clinical data, independently reviewed the radiographs. The questionable cases were discussed to reach an agreed diagnosis. Vertebral fractures (VFx) were diagnosed on visual inspection using the semi-quantitative visual assessment described by Genant et al[44]. The spine deformity index (SDI) was successively calculated according to Eller-Vainicher et al[45] and Crans et al[46].

### Balance assessment

In all patients balance was assessed by the Romberg test, which was performed according to the usual clinical practice[47].

### Artificial neural network analysis

The outline of the relationships among all the studied parameters was investigated by an analysis based on artificial neural networks. A mapping method, described in detail elsewhere[45,48–53] was used to graphically highlight the most important links between variables, using the algorithm Auto Contractive Map, a particular type of neural network capable of highlighting the bearing structure of the data base with the most important associations between the study variables. This network, after a learning phase in which all the variables are interconnected in a dynamic way, builds a matrix of weights whose values are proportional to the strength of the associations between the variables. The weights are then transformed into physical distances. The variable pairs with higher weights of connections are put closer in the semantic map, and vice versa. A mathematical filter represented by the minimum spanning tree (MST) is applied to the matrix of distances and generates a graph. This step allows the observation of general wiring diagrams between the variables and the detection of variables that act as "hubs", being highly connected. This matrix of connections, as detailed by Buscema and Grossi[48,49], retains the non-linear associations between variables and captures the connection diagrams between clusters. From a mathematical point of view, the positioning of the connections is equal to the ranking of the joint probability between each variable and the others. Each continuous variable for which a cut-off paradigm was not available has been transformed into two complementary variables. For this purpose the values of the variable have been scaled from 0 to 1 and a complementary variable has been obtained by subtracting its value scaled by 1. Therefore two classes of variables are formed: a class which shows values in the high range and a class that highlights values in the lower range. In the map these two complementary forms have been named as high and low. This scaling pre-processing is required to make a comparison proportionally among all the variables, and to understand the system of each variable when the values tend to be high or low. This is important, because in non-linear systems the position of the high and low values of a given variable is not necessarily symmetrical.

Maximally Regular Graph (MRG) has been used to delineate relationships between variables in patients with SDI >5 (VFx yes) and with SDI<5 (VFx no). MRG shows the maximal intrinsic complexity of the map by including the highest number of cyclic regular microstructures between the variables, as elsewhere described[49].

## Results

The personal and anthropometric data, SDI, biochemical bone markers, quantitative and qualitative bone parameters are expressed in Table 1 as mean, standard deviation, median and range.

**Table 1:** Characteristics of patients.

	MEAN	SD	MEDIAN	MAX	MIN
**AGE (yrs)**	67.61	10.80	68.25	87.81	45.43
**YRS from menopause**	18.86	11.13	19.15	46.29	0.36
**BMI (kg/m2)**	24.86	3.89	24.49	42.46	16.89
**SDI**	2.03	4.05	0.00	19.00	0.00
**ALP (U/L)**	72.98	23.96	68.00	158.00	30.00
**BAP (U/L)**	48.97	12.23	48.00	85.00	17.00
**CTX (pg/dl)**	484.08	323.24	425.70	2674.00	63.65
**25 vitamin D (ng/ml)**	25.27	11.72	24.20	74.40	4.00
**BMD Lumbar (g/cm^**2**^)**	0.79	0.15	0.77	1.43	0.48
**BMD Neck (g/cm^**2**^)**	0.59	0.09	0.58	0.97	0.41
**TBS**	1.15	0.12	1.16	1.50	0.82
**BS Lumbar**	4.06	2.21	3.59	11.95	0.49
**T-score neck**	-1.88	0.78	-1.90	0.10	-3.60
**Z-score neck**	-0.47	0.94	-0.60	1.70	-2.50

Legend. ALP: alkaline phosphatase. BAP: alkaline phosphatase bone isoenzyme. CTX: type I procollagen N-terminal telopeptide. BMD: lumbar spine bone mineral density. TBS: trabecular bone score. BS: bone strain. SDI: spine deformity index. BMI: body mass index.

**Fig 1:**
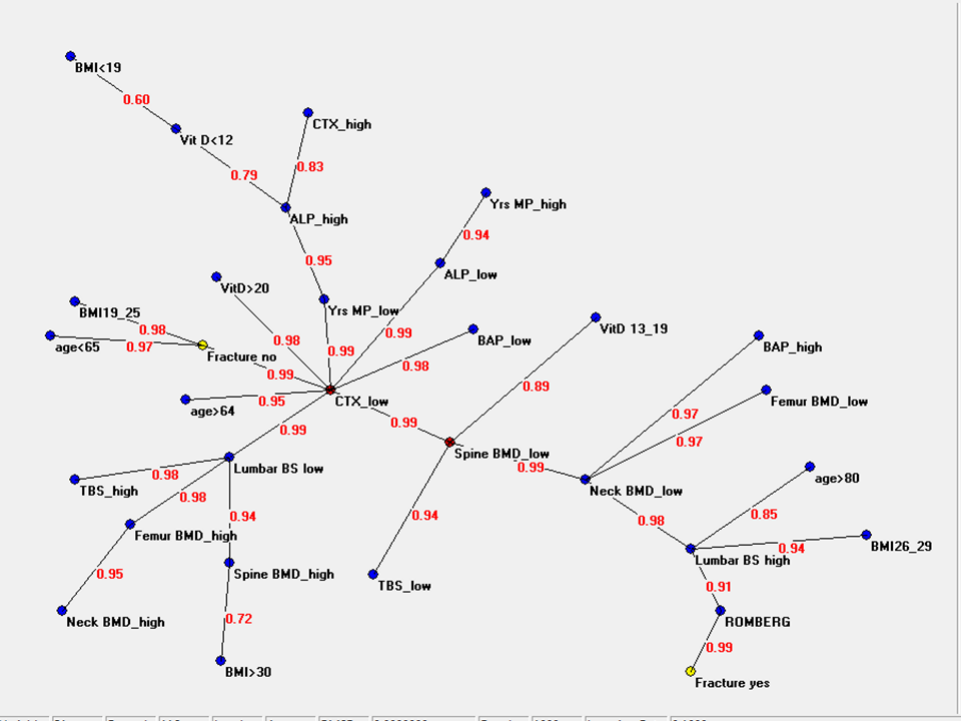
Semantic map showing the relations between the investigated anagraphic, antropometric, densitometric, biochemical and clinical parameters.

Fig 1 shows the connectivity map (MST) of all variables linked to fracture status and the multiple variables linked to the osteoporotic condition, including quantitative and qualitative bone parameters, bone turnover markers, Romberg test. Semantic map resembles to be divided in two parts. The first spreads around a node that appears as a hub, the “CTX low” variable, to whom bone status parameters, bone markers, anagraphic data, fracture status are directly connected. The second part develops along a way that, through bone status parameters and balance condition, drives to fracture. [Fig 1. Semantic map showing the relations between the investigated anagraphic, antropometric, densitometric, biochemical and clinical parameters. Legend. ALP: alkaline phosphatase. BAP: alkaline phosphatase bone isoenzyme. CTX: type I procollagen N-terminal telopeptide. BMD: lumbar spine bone mineral density. TBS: trabecular bone score. BS: bone strain. SDI: spine deformity index. BMI: body mass index.]

**Fig 2:**
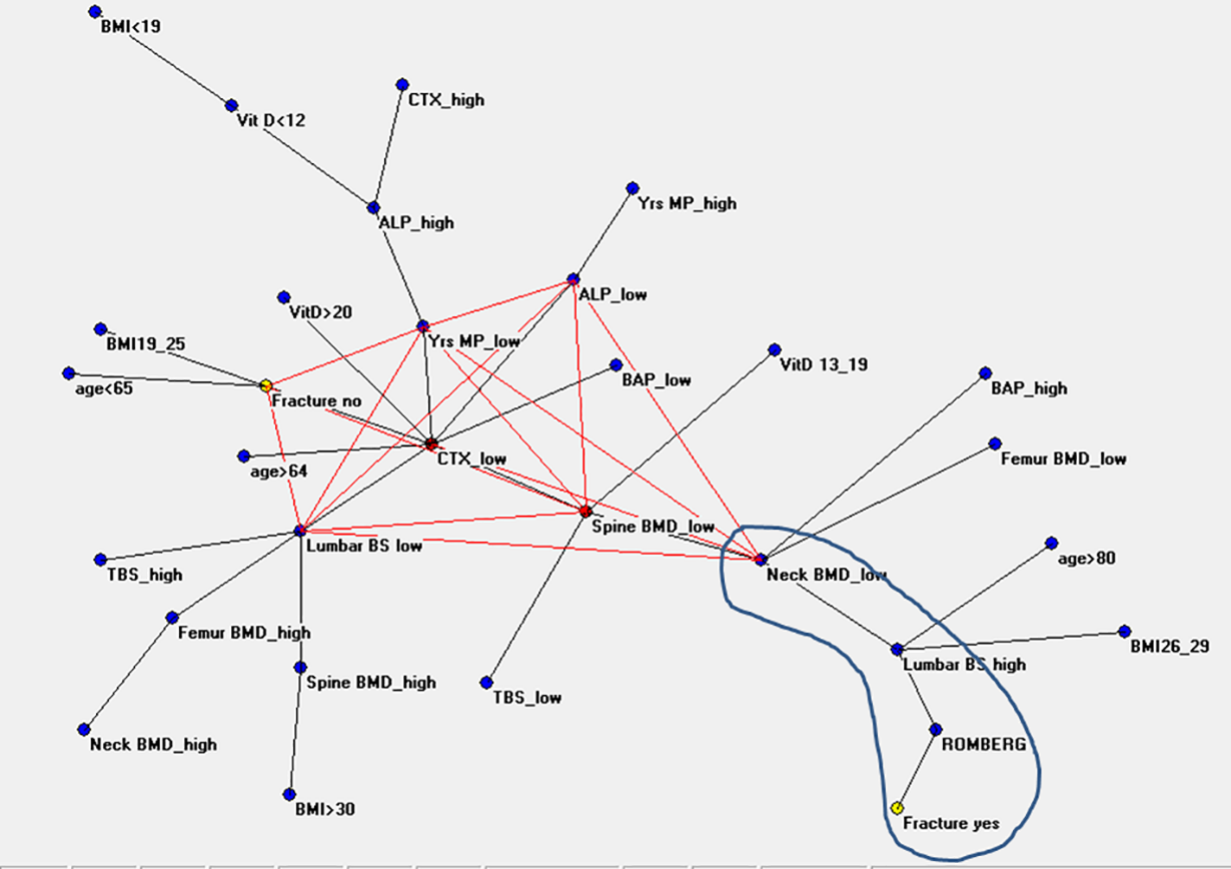
Maximal Regular Graph of the investigated anagraphic, antropometric, densitometric, biochemical and clinical parameters.

Fig 2 shows the MRG and the interconnections of those parameters that lead patients to a VFx event (“Fracture yes”). It is to notice that the above cited first part of the map presents the rich interconnections between the hub “CTX low” and its linked variables, while in the second part the scant variables are very poorly interconnected. [Fig 2 Maximal Regular Graph of the investigated anagraphic, anthropometric, densitometric, biochemical and clinical parameters. Legend. ALP: alkaline phosphatase. BAP: alkaline phosphatase bone isoenzyme. CTX: type I procollagen N-terminal telopeptide. BMD: lumbar spine bone mineral density. TBS: trabecular bone score. BS: bone strain. SDI: spine deformity index. BMI: body mass index.]

## Discussion

This study aims to contribute to the debate about the question of overdiagnosis and consequent overtreatment of osteoporosis. The debate arose regarding the identification of the patients who may gain the greatest advantage from treatment in order to avoid fragility fractures, and has got particular interest after Jarvinen’s articles, not yet finding a solution.

In this study old postmenopausal women were recruited, representative of the normal old female population, with a median age close to seventy years and a Gaussian distribution of BMI.

This population shows a wide distribution of CTX values, as expected from the high inter-individual variations of the osteoclastic bone resorption[54,55].

Mean and median vitamin D values of the population appear in the normal range according to Institute of Medicine (IOM)[56], but with a wide spread of the values reflecting both naïve and treated vitamin D condition of a community dwelling elderly population like the one examined.

Regarding bone mass, the women present an osteopenic condition, with a TBS, index of the spatial distribution of the trabeculae, below the value considered as the normal cutoff for a French population, which is geographically near to our region and of the same ethnicity[57].

Analysis of data was performed by the means of artificial neural network analysis, an adaptive mathematical model particularly suitable for analyzing non linear interactions among a high number of clinical variables. It has been widely used in various chronic pathological conditions [45,51–53,58–60]. Moreover, differently from standard statistical tests, ANNs is a valid mathematical tool for the study of small sized samples with unbalance between variables and records [59,60].

In medical field ANNs data mining represents a relatively new philosophy emerging with the advent of genomic and functional data. The available techniques offered by classical statistics like Principal Component Analysis of Hierarchical clustering suffer from a number of drawbacks due to the complexity of possible interactions between risk factors, their non-linear influence on the disease occurrence and the considerable stochastic components.

The more common algorithms of linear projections of variables require generally a Gaussian distribution of data and have limited power when the relationships between variables are non linear. Application of these methods may lose important informations, and establishing precise associations among variables having only the contiguity as a known element is difficult. Another limitation of currently used statistical methods is that mapping is generally based on a specific kind of “distance” among variables (e.g. Euclidean, City block, correlation, etc) and gives origin to a “static” projection of possible associations. In other words, the intrinsic dynamics due to active interactions of variables in living systems of the real world is completely lost.

Auto-Cm system arises just to overcome these limitations. The mathematics of Auto-Cm has been described in detail elsewhere [48]. Auto-CM has been efficiently applied in many different medical complex contexts with very interesting results, like Alzheimer disease and dementia, gastrointestinal reflux, non variceal upper gastrointestinal bleeding, autism [49,58–60], helping clinicians in discerning the most significant features of diseases with multifactorial aspects.

ANNs of the data of the patients’ population is graphically shown as a semantic map in Fig 1. Bone resorption marker “CTX low”, indicating a scant remodeling status, appears to be the central hub of the connections of most variables regarding bone status, as well as bone metabolism, patients characteristics and fracture status (“Fracture no”).

Normal vitamin D is directly connected with the hub, suggesting that in older patients a good vitamin D status characterizes the condition of <good health>, that is to say absence of fracture despite low bone density. This is in accordance with the recent considerations in literature, namely that vitamin D could represent a marker of health status[61,62].

Parameters of good bone quality, indicated by “TBS high” and “BS low”, are connected with the hub “CTX low”, index of low bone turnover; in particular, “BS low” is connected directly. Also the parameters of good bone quantity, namely “neck, femur and spine BMD high”, are connected to the hub, but through “lumbar BS low”, indicator of good bone quality, as already said.

The semantic map shows another interesting aspect. Spine and neck low BMD and impaired balance, represented by a positive Romberg test, are one way connected to fracture status (“Fracture yes”) suggesting a strict correlation between balance and fracture risk.

As shown in Fig 2, the variables indicating a recent menopause (“Yrs MP low”), a low bone turnover (“CTX low”, “ALP low” and its bone isoenzyme low), a low bone mass both of spine and of femur (“Spine BMD low” and “Neck BMD low”), a good bone quality (“Lumbar BS low”) and the absence of fracture (“Fracture no”), present a high density of interconnections. This evidence could suggest that in a postmenopausal population, where a low bone mass is obviously expected, the presence of low bone turnover and good bone quality makes fracture unlikely. The complex interconnections between the variables related to the state of absence of fracture (good bone quality, low bone turnover) include normal serum vitamin D, that is directly related to the hub “CTX low”, like the cited variables. On the other side of the semantic map, namely that of “Fracture yes”, one can easily observe a simplification of the connections between the variables and a scant number of involved variables. Here, poor bone quantity and quality, as well as an impaired balance, lead directly to fracture.

So, utilizing the statistic method of ANN analysis, this study seems to suggest that in the elderly population the conditions of reduced bone mass and insufficiency/deficiency of vitamin D, which are frequently noticed, may not appear to represent a relevant discriminating factor for the pharmacological treatment of osteoporosis. These conditions are not connected to the fracture event, in contrast to the impaired bone quality and the deficient balance. In fact, these last aspects, that are really closed to fracture event in the map, appear to be the true conditions that would require a treatment. Being poor bone quality not yet amenable of treatment, impaired balance should be investigated and ameliorated. However, the execution of the Romberg test, although very simple to perform, is not usually indicated in the guidelines of osteoporosis, whereas its positivity could allow the clinicians to prescribe a rehabilitation program that possibly would lead to an improvement of balance. This program could bring to a reduction of falls, that are, together with bone fragility, the real determinants of fractures, especially in frail older people.

Our work presents some limitations. In the pre-existing data collection of this ancillary study the number of falls was not available. Indeed, the Romberg test is a valid and simple method to recognize patients prone to falls and consequently exposed to fracture risk. Another limitation could be the non-quantitative nature of the Romberg test and, therefore, it suffers from the subjective operator’s judgement in the patient’s classification. However, it is widely and usefully utilized in clinical general practice. Moreover, the not very large number of patients could be a limitation, but artificial neural network analysis is a tool that can deal with complexity, even if the population sample is small and with unbalanced proportion between variables and records. So, ANNs is able to pass the matter of sample dimension [59].

In conclusion, the connections evidenced by the ANNs analysis suggest three steps that could be relevant in the evaluation of an elderly population at high risk of fracture and amenable of treatment: 1. a DXA examination for determining bone quantity and quality; 2. a blood CTX test in order to examine bone turnover; 3. the Romberg test for balance assessment.

Proper further studies will reveal if these ANNs’ interesting connections may be relevant also in actual clinical practice to improve the pertinence of medical interventions for osteoporosis management.

## Supporting information

S1 File Minimal data set. This file contents a minimal set of data in excel format.
(XLSX)
